# Nonlocality, Superposition, and Time in the 4+1 Formalism

**DOI:** 10.3390/e25111493

**Published:** 2023-10-29

**Authors:** Filip Strubbe

**Affiliations:** Department of Electronics and Information Systems, Ghent University, Tech Lane Ghent Science Park-Campus A 126, 9052 Ghent, Belgium; filip.strubbe@ugent.be

**Keywords:** quantum gravity, nonlocality, EPR, measurement problem, arrival time problem, problem of time, arrow of time, superposition

## Abstract

The field of quantum gravity struggles with several problems related to time, quantum measurement, nonlocality, and realism. To address these issues, this study develops a 4+1 formalism featuring a flat 4D spacetime evolving with a second form of time, τ, worldlines that locally conserve momentum, and a hypersurface representing the present. As a function of τ, worldlines can spatially readjust and influences can travel backward or forward in the time dimension along these worldlines, offering a physical mechanism for retrocausality. Three theoretical models are presented, elucidating how nonlocality in an EPR experiment, the arrival time problem, and superposition in a Mach–Zehnder interferometer can be understood within this 4+1 framework. These results demonstrate that essential quantum phenomena can be reproduced in the 4+1 formalism while upholding the principles of realism, locality, and determinism at a fundamental level. Additionally, there is no measurement or collapse problem, and a natural explanation for the quantum-to-classical transition is obtained. Furthermore, observations of a 4D block universe and of the flow of time can be simultaneously understood. With these properties, the presented 4+1 formalism lays an interesting foundation for a quantum gravity theory based on intuitive principles and compatible with our observation of time.

## 1. Introduction

In the last hundred years, physics has maneuvered itself into an uncomfortable position. On the one hand, quantum mechanics and general relativity are highly successful theories in their respective domains, having passed the stringest experimental tests. On the other hand, there are several persisting fundamental problems when attempting to unify general relativity with quantum mechanics. This is largely due to an incompatibility between the notions of time in quantum mechanics and in general relativity, referred to as the problem of time [1,2,3], and due to problems with how measurements happen in quantum theory, referred to as the measurement problem [4,5,6,7]. Additionally, as a consequence of bizarre features of quantum mechanics, it has become hard to understand reality in an intuitive way. For example, EPR experiments have demonstrated that local realism must be abandoned in any description of reality [8,9,10,11,12,13,14,15]. Here, locality is the concept that particles are influenced only by their immediate surroundings, and realism is the notion that there is an objective reality existing independently of observation. Both concepts seem indispensable for such an intuitive view of reality. And lastly, there is still no satisfying explanation for the observation of the present and of the arrow of time [16,17,18]. While the quantum gravity community remains optimistic about resolving these issues within leading theories like string theory or loop quantum gravity, this work argues that these problems must be solved at the foundations before a satisfying theory of quantum gravity can be constructed.

This work aims to develop a basis for a theory of quantum gravity free of the above-mentioned foundational issues. To achieve this, a secondary form of time, τ, is added to flat four-dimensional spacetime to produce an evolving block universe, referred to as the 4+1 formalism (see Section 2). In this 4+1 framework, particles are exclusively described by means of worldlines that can spatially reorient and can transmit interactions (backward and forward in the time dimension) as a function of τ. And a hypersurface Σ representing the present is invoked that shifts in the time dimension as a function of τ. The general concept of adding an evolution parameter τ to 4D spacetime was first developed in Stueckelberg–Horwitz–Piron theory [19,20] and analyzed in the works of Land [21,22,23]. There are also similarities with the evolving block universe introduced by Broad [24] (see also [25]) and the crystallizing block universe of Ellis and Rothman [26], with the difference that in this work explicit reference is made to an evolution parameter τ.

Three case studies are presented in Section 3, illustrating how essential quantum phenomena can be reproduced within this 4+1 formalism. Firstly, a model for an EPR experiment is developed and simulated in Section 3.1, showing how entanglement and nonlocality can be understood. This model is an implementation of the idea first proposed by Costa de Beauregard of influences traveling in a zigzag way along worldlines, forward and backward in time [27,28,29]. Secondly, an isotropic source with hemispherical detectors is modeled in Section 3.2 to discuss quantum measurement and the arrival time problem. And thirdly, a model for a Mach–Zehnder interferometer is developed in Section 3.3 to show how superposition and wave–particle duality can be understood in the 4+1 formalism.

Section 4 discusses several intriguing features of the 4+1 formalism and how it resolves or circumvents the aforementioned issues. Perhaps most importantly, it shows how quantum phenomena can be reproduced while adhering to principles of locality, determinism, and realism at a fundamental level beyond ordinary 4D spacetime. This may allow us to understand reality in a more intuitive way than is possible within the confines of ordinary spacetime. Additionally, due to its worldline-based approach, it offers a solution for the measurement problem, a sensible framework for particle interactions satisfying local momentum conservation, and a natural explanation for the quantum-to-classical transition. Furthermore, the combination of its two forms of time, namely, a time dimension *t* and an evolution parameter τ, with the shifting hypersurface Σ, allows for understanding both the observation of a 4D block universe and of the flow (and arrow) of time. Also, implications of the interferometer model for laboratory tests of quantum gravity are discussed.

Ultimately, applying the same 4+1 principles to curved spacetime and formulating a more comprehensive worldline-based alternative for quantum mechanics may lead to a unified framework capable of explaining both quantum mechanical and gravitational phenomena. In this way, the presented 4+1 formalism shows an interesting path towards a theory of quantum gravity.

## 2. Theory

### 2.1. The 4+1 Formalism

The 4+1 formalism consists of a one-parameter family of 4D spacetimes, labeled with the parameter τ. This parameter τ is a scalar (τ∈IR) that acts like a second form of time. For each value of τ, there is an associated 4D spacetime M(τ). Symbolically, this can be represented as an M×IR construction. It differs from a five-dimensional structure in which there would be extra space or time coordinates. The basic concept of such a 4+1 formalism, namely, the addition of an evolution parameter τ to 4D spacetime, was first developed by Stueckelberg, Horwitz, and Piron [19,20]. Recent advances in the 4+1 formalism were made by Land [21,22,23].

**Figure 1:**
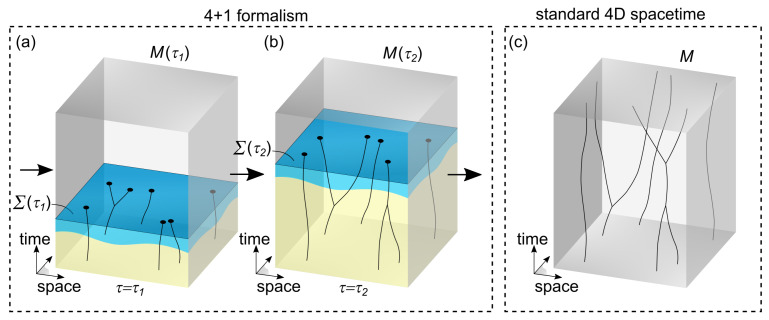
Illustration of the 4+1 formalism used in this work. In (**a**), at $\tau =\tau_{1}$, the flat 4D spacetime $M(\tau_{1})$ contains worldlines in the past region from the hypersurface $\Sigma (\tau_{1})$. In the yellow region, worldlines have largely reached a fixed, crystallized configuration. But in the light-blue region close to $\Sigma (\tau_{1})$, there are still dynamics of worldlines as a function of τ, orchestrated using evolution laws. In the future region with respect to $\Sigma (\tau_{1})$, there are no worldlines but only elementary physical entities (not shown). In (**b**), at $\tau =\tau_{2}$, the hypersurface $\Sigma (\tau_{2})$ has shifted into the positive time dimension and worldlines have adjusted accordingly. A 4D spacetime $M(\tau_{2})$ is produced that may differ from $M(\tau_{1})$, even at times *t* corresponding to the past region of $\Sigma (\tau_{1})$. Also, a larger part of spacetime has become fully crystallized (yellow region). The τ-dynamics occurring close to the crystallization front (blue region) are essential for reproducing quantum phenomena. By associating the shifting hypersurface Σ(τ) with the present, our observation of the flow and arrow of time can be explained. The fully crystallized region of spacetime for τ→∞, which reflects our macroscopic observations, corresponds to a classical 4D spacetime, as shown in (**c**).

This work is restricted to flat 4D spacetimes M(τ) and introduces additional elements (see Figure 1a,b). Each spacetime is then labeled with orthogonal coordinates (x,y,z,t) that are properly normalized using rulers and clocks. The connection between these spacetimes is established by considering physical entities existing in each spacetime associated with a different value of τ. Ignoring any microscopic degrees of freedom associated with these physical entities, each entity is characterized by a τ-dynamic event $x^{\mu }\left(\tau \right)$, where the coordinates $x^{\mu }$ for μ=0,1,2,3 correspond, respectively, to x,y,z,t. To produce a physically meaningful universe, it is imposed that the invariant proper length $\Delta s^{2}=\Delta x^{2}+\Delta y^{2}+\Delta z^{2}-c^{2}\Delta t^{2}$ (with speed of light *c*) between any two physical events in each spacetime varies continuously as a function of τ, satisfying $\lim_{\tau \to \tau_{1}}\Delta s^{2}\left(\tau \right)=\Delta s^{2}\left(\tau_{1}\right)$. Additionally, the coordinates of each spacetime are chosen such that also the coordinate values $x^{\mu }\left(\tau \right)$ of physical events vary continuously as a function of τ, satisfying $\lim_{\tau \to \tau_{1}}x^{\mu }\left(\tau \right)=x^{\mu }\left(\tau_{1}\right)$. A preferred frame of reference is established via a set of physical events that carry the same coordinates in each spacetime.

Furthermore, this work describes particles solely by means of (one or multiple) τ-dynamic worldlines rather than with wave functions or fields. A general expression for a τ-dynamic worldline is $x^{\mu }\left(\lambda ,\tau \right)$, where τ selects the 4D spacetime M(τ) and λ is an affine parameter describing the worldline in that spacetime as usual. Within each spacetime, all worldlines obey local conservation of momentum.

And finally, a hypersurface Σ(τ) is introduced in each spacetime characterized by a constant time $t_{e}\left(\tau \right)=\beta \tau$ in the preferred frame. Since β is a positive scalar, this hypersurface shifts in the positive time dimension as τ increases monotonously.

With the above assumptions, a 4+1 formalism is established with a trivial connection between different flat spacetimes. This connection enables us to compare coordinates of events in different spacetimes in a physically meaningful way. For all practical purposes, one may interpret this 4+1 construction as a single, flat 4D spacetime in which the dynamics of worldlines are described as a function of τ. This emphasizes that τ is envisioned as an evolution parameter for a flat 4D spacetime similar to how *t* is the evolution parameter for 3D space in classical mechanics. Dynamics as a function of τ are referred to as “τ-dynamics” to distinguish these from usual dynamics as a function of time *t*.

### 2.2. The Present and the Flow of Time

The hypersurface Σ(τ) is introduced for explaining our observation of the present and of the flow of time (or the arrow of time). This hypersurface (blue surface in Figure 1a,b) separates a past region filled with τ-dynamic worldlines from a future region in which worldlines are not yet formed. Since the hypersurface Σ(τ) is the only physically relevant structure that points at specific time coordinates, it is implied that Σ(τ) corresponds to the present in each 4D spacetime associated with a value of τ. As τ increases, Σ(τ) shifts in the positive direction of the time dimension in order to explain the flow and the arrow of time.

Seeing τ as an external clock governing changes in 4D spacetime is relevant for making sense of this observation of the flow of time. In contrast, when assuming that τ represents the coordinate of yet another dimension such that all associated 4D spacetimes exist in a single 5D block universe, one again arrives at a static configuration which apparently lacks such ability to explain the flow of time.

Let us analyze the two regions with respect to Σ(τ) in more detail. The past region is populated by worldlines that are τ-dynamic and can locally interact with each other. To produce a framework consistent with our observations, it is assumed that sufficiently far in the past from Σ(τ), worldlines become largely constrained by their evolution laws, such that in good approximation they become fixed as a function of τ. Therefore, in the far past (yellow region in Figure 1a), a crystallized region is obtained that can be described in good approximation as a part of a fixed spacetime *M*. As τ increases and Σ(τ) shifts in the positive time direction, worldlines grow and adjust as determined via their evolution laws. Also, a larger part of spacetime becomes crystallized (extended yellow region in Figure 1b). Hence, in the limit for τ→∞, a completely crystallized spacetime is obtained that can be described with a standard spacetime *M*. Figure 1c illustrates this standard spacetime *M*. In the future region with respect to Σ(τ), worldlines are not yet fully formed, but the necessary physical entities to construct worldlines are already present there. The hypersurface Σ(τ) itself collects the end events of all worldlines from the past region that reach it (marked with black dots in Figure 1a,b).

### 2.3. Observations in the 4+1 Formalism

Since standard observers, particles, and measurement devices are all made from τ-dynamic worldlines, measurements and observations follow from interactions between such worldlines. For example, after a primary interaction between two worldlines, there can be a whole cascade of further interactions and τ-dynamics that ultimately cause a detector to settle in a specific output (pointer) state. Importantly, worldlines and their τ-dynamics cannot be directly observed. Observers can only observe the present, characterized by the endpoints of worldlines at the hypersurface Σ(τ). To motivate this, it is assumed that these endpoints carry physical properties (marked with black dots in Figure 1a,b) that are relevant for the observation of the present. Since τ-dynamics cannot be observed directly, measurements reflect (per definition) only the equilibrium configuration of worldlines, which in turn corresponds to the fully crystallized spacetime *M* (as illustrated in Figure 1c).

With its two forms of time, namely, a time dimension *t* and an evolution parameter τ, and its shifting hypersurface Σ(τ), the 4+1 formalism can simultaneously explain why observations agree with 4D spacetime and with the flow of time. On the one hand, using standard measurement tools, the coordinates (x,y,z,t) (or $x^{\mu }$ in short) of interaction events can be determined by an observer. Here, x,y,z are spatial coordinates that can be determined using rulers, and the value of *t* can be determined indirectly using a clock, which is in this context yet another device made from τ-dynamic worldlines. Observers can map out measured events $x^{\mu }$ to form a 4D spacetime $M_{exp}$. As τ increases, more events are collected and an increasing part of this 4D spacetime is gradually built up. This experimentally obtained spacetime $M_{exp}$, pieced together from our observations, is identical to the fully crystallized 4D spacetime *M* (illustrated in Figure 1c) down to experimental precision. And it explains why our observations seem to be limited to four coordinates (x,y,z,t) such that the second time τ becomes effectively hidden. But on the other hand, the hidden evolution parameter τ and the shifting hypersurface Σ explain why we collect these events in a particular way. Namely, as τ increases, observations are made of the form (x(τ),y(τ),z(τ),t(τ)), where t(τ) increases together with the time $t_{e}\left(\tau \right)=\beta \tau$ of the hypersurface Σ(τ). This is in agreement with our observation of the flow and of the arrow of time. Furthermore, by simply relabeling τ with *t*, the coordinates (x(τ),y(τ),z(τ)) in the preferred frame can be interpreted as (x(t),y(t),z(t)). As such, the usual situation of classical mechanics is retrieved in which spatial coordinates depend on an evolution parameter *t*.

### 2.4. Evolution Laws

Close to the hypersurface Σ(τ) (light-blue region in Figure 1a,b), worldlines may still have a remaining dynamic freedom as a function of τ, orchestrated via evolution laws. Ultimately, it is desired to develop universal evolution laws for how worldlines behave as a function of τ that replace the standard quantum formalism. In this work, only for the specific case studies in Section 3, evolution laws are developed. The τ-dynamics of worldlines considered here include spatial reorientations and internal changes allowed by local conservation of momentum in each spacetime M(τ). When τ increases and Σ(τ) shifts forward in the time dimension, the situation in the past region of Σ(τ) changes, for example, because the spatial lengths of worldlines increase or because of newly formed interactions between worldlines. These new circumstances may lead to a different optimal worldline configuration according to the evolution laws. As a result, there is a continuous readjustment of worldlines in the past region from Σ(τ) as a function of τ imposed by the evolution laws. They remain τ-dynamic until their evolution laws lock them into a final configuration. Importantly, this means that changes can occur as a function of τ such that $M(\tau_{2})$ differs from $M(\tau_{1})$ (with $\tau_{2}>\tau_{1}$), even in the past region with respect to $\Sigma (\tau_{1})$. In this way, the τ-dynamics of worldlines implement a physical mechanism for retrocausal action in 4D spacetime.

In principle, there is no restriction for how far into the past of Σ(τ) that evolution laws can have an influence. In the case of EPR experiments, this may amount to very long times. However, in most cases, worldlines are expected to quickly reach a fixed optimal configuration. For example, in a double-slit experiment, the spatial adjustments of worldlines occur in the near field typically in the order of femtoseconds. So, overall, we can assume that important τ-dynamics only play in a very small region of spacetime in the past of Σ(τ), whereas the further past region quickly settles into a fixed equilibrium configuration. The rate at which τ-dynamics occur is finite but in principle unrestricted. This means that one may choose worldlines to reorient spatially and interactions to travel along worldlines arbitrarily fast (or slow) as a function of τ. However, to agree with the experimental observations of specific phenomena, such as the EPR experiment analyzed in Section 3.1, a minimal rate of these τ-dynamics may be required.

### 2.5. Quantum Phenomena

Classical observations can easily be explained in the 4+1 formalism. This simply requires prohibiting the τ-dynamics of worldlines in the past region of Σ(τ). In this case, as Σ(τ) shifts in the positive time dimension, more of a fixed 4D spacetime is revealed. And in such a standard spacetime, any classical effect based on locally interacting worldlines can be explained. But quantum phenomena can in principle also be reproduced in the 4+1 formalism. For example, in a previous work, it has been demonstrated how double-slit interference can be understood in the 4+1 formalism [30], and in Section 3, three more quantum phenomena are analyzed. As can be expected, the τ-dynamics of worldlines are crucial for explaining such quantum phenomena in the 4+1 formalism.

### 2.6. The Measurement Problem

Quantum mechanics relies on the deterministic evolution of the wave function followed by a collapse mechanism to explain determinate outcomes of a measurement [5]. The measurement problem refers to conflicts with relativity and with conservation of momentum related to this wave function collapse [7]. The three quantum phenomena analyzed in Section 3 demonstrate different aspects of the measurement problem. EPR experiments (see Section 3.1) have proven that correlations between outcomes of measurements on entangled particles cannot be explained with any theory relying on local realism [8,9,10,11,12,13,14]. As a result, EPR correlations arise seemingly instantaneously across large spatial distances when a measurement is performed. And this is problematic because it seems to require interactions exceeding the speed of light and because instantaneity is ill defined in relativity. In some experimental configurations, it is not even clear if the measurement on one particle of an entangled pair occurs before the other or oppositely, leading Bancal et al. to remark that this “gives further weight to the idea that quantum correlations somehow arise from outside spacetime, in the sense that no story in space and time can describe how they occur” [9]. Hence, this suggests that something may be wrong with the standard concept of spacetime. The isotropic emission of a particle in Section 3.2 focuses on the arrival time problem in a quantum measurement. And issues with momentum conservation in quantum measurements are analyzed in the Mach–Zehnder interferometer (see Section 3.3).

The developed 4+1 formalism deliberately replaces the standard quantum formalism with a formalism based on τ-dynamic worldlines to overcome the measurement problem. Then, worldlines and their interactions determine the outcome of each experiment, without requiring instantaneous collapse. And, by construction, momentum is locally conserved.

### 2.7. Realism, Locality, and Determinism in the 4+1 Formalism

The results of quantum theory have led many physicists to believe that reality is impossible to comprehend with common sense and that we must accept that nature is fundamentally based on features like nonlocality, superposition, and uncertainty. This general feeling was expressed by physicists like Heisenberg [31] and Feynman [32] and is supported by a large majority today. However, it is important to realize that claims about what quantum mechanics tells us about reality usually rely on tacit assumptions like four-dimensional spacetime that may be flawed. Therefore, by changing our concept of spacetime, it may be possible to recover intuitive concepts like realism, locality, and determinism at a more fundamental level of reality. The 4+1 formalism developed in this work aims to achieve this by introducing a second form of time and by relying solely on worldlines rather than wave functions or fields.

Since the 4+1 formalism goes beyond ordinary spacetime, one must be careful with typical definitions of realism, locality, and determinism that (tacitly) assume a standard spacetime. To avoid such confusion, this work adopts the usual definitions of locality and realism when assuming the perspective of ordinary 4D spacetime:

- Locality: the concept that particles, seen as objects existing in ordinary 4D spacetime, are only influenced by their immediate surroundings and can only influence each other by means of interactions limited by the speed of light.
- Realism: the concept that particles, seen as objects existing in ordinary 4D spacetime, have defined properties even before measurement.
- Determinism: the concept that every event in spacetime can only be causally influenced by events in its past light cone.

However, different definitions are adopted when going beyond the limiting assumption of ordinary 4D spacetime, like in the presented 4+1 formalism:

- Fundamental locality: The concept that particles, at their most fundamental level, are only influenced by their immediate surroundings. Note that here the meaning of the term “surroundings” may go beyond the usual concept in 4D spacetime.
- Fundamental realism: the concept that particles, at their most fundamental level, have defined properties even before measurement.
- Fundamental determinism: determinism as a function of the evolution parameter τ.

As a consequence, even if observations may appear nonlocal from the perspective of ordinary spacetime, the underlying mechanism in the 4+1 formalism may still be fundamentally local. Similarly, even if realism seems hopeless from within the confines of ordinary spacetime, there may still exist fundamental realism at a deeper level of reality.

## 3. Results

The 4+1 framework presented in Section 2 relies entirely on τ-dynamic worldlines. This means that evolution laws for these worldlines must be developed to replace the standard quantum formalism. In this section, theoretical models are developed which explain three key quantum phenomena in the 4+1 formalism: EPR nonlocality, the arrival time problem, and superposition in a Mach–Zehnder interferometer.

### 3.1. EPR Nonlocality in the 4+1 Formalism

EPR experiments demonstrate one of the most surprising features of quantum mechanics, namely, quantum nonlocality. Let us consider a typical EPR configuration with polarization-entangled photon pairs represented by the singlet state [33,34]:

(1) $$|\Psi \rangle =\frac{1}{\sqrt{2}}\left({|H\rangle }_{A}{|H\rangle }_{B}+{|V\rangle }_{A}{|V\rangle }_{B}\right)$$

where $|H\rangle$ and $|V\rangle$ are, respectively, the horizontal and vertical polarization and subscripts *A* and *B* refer to Alice’s and Bob’s photons. Measurements can be performed by Alice and Bob on their respective photons in spatially separated regions. For example, they can choose different measurement settings by rotating a polarizing beamsplitter (PBS) and can detect if a photon passes (outcome *A* and *B*) or is deflected (outcome $\bar{A}$ and $\bar{B}$). If we assume that Alice measures first with PBS $P_{A}$ set at an orientation $\theta_{A}$, then quantum theory suggests that the quantum state of Bob’s photon “instantaneously” adapts to Alice’s measurement result. If the photon passes through PBS $P_{A}$ such that Alice observes outcome *A*, the entangled system collapses into a state where both photon *A* and photon *B* have a polarization along $\theta_{A}$. In the opposite case that Alice observes outcome $\bar{A}$, the polarization state of both photons collapses perpendicularly to $\theta_{A}$. After this, when Bob decides to measure his photon using his PBS $P_{B}$ with chosen orientation $\theta_{B}$, he either observes that the photon passes (outcome *B*) or is deflected (outcome $\bar{B}$). Quantum theory predicts the following probabilities for joint detection at *A* and *B* [34]:

(2) $$\begin{array}{c} P\left(A,B\right)=P\left(\bar{A},\bar{B}\right)=\frac{\cos^{2}\left(\theta_{A}-\theta_{B}\right)}{2}\\ P\left(A,\bar{B}\right)=P\left(\bar{A},B\right)=\frac{\sin^{2}\left(\theta_{A}-\theta_{B}\right)}{2} \end{array}$$

This illustrates that the outcome of Bob’s experiment depends on the measurement setting of Alice, no matter how far they are separated (and vice versa). This is surprising since it seems to require an interaction going faster than light. These predictions of quantum theory have been confirmed in various EPR experiments [9,10,15]. According to Bell’s theorem, the obtained quantum correlations cannot be explained with any hidden variable theory based on local realism [35]. Since also a large class of nonlocal realist theories has been ruled out, it seems that realism is increasingly at risk [8]. Furthermore, even though quantum nonlocality does not allow faster-than-light communication and does not violate relativity in this sense, there is still the difficulty of understanding the measurement process of the EPR experiment in a relativistic setting [9,36].

Next, we analyze whether one can make more sense of the EPR paradox in the 4+1 formalism.We follow up on the idea first proposed by Costa de Beauregard of influences traveling along worldlines, back to a common interaction event at an earlier time coordinate [27,28,29]. This results in a relaxed measurement independence, which can be exploited to reproduce the nonlocal correlations of EPR experiments [37,38,39]. However, like any retrocausal idea, also the idea of Costa de Beauregard conflicts with the causality of standard spacetime and is in need of a second form of time. Therefore, here, the idea of influences traveling along worldlines is embedded in the 4+1 formalism which features such a second time τ.

**Figure 2:**
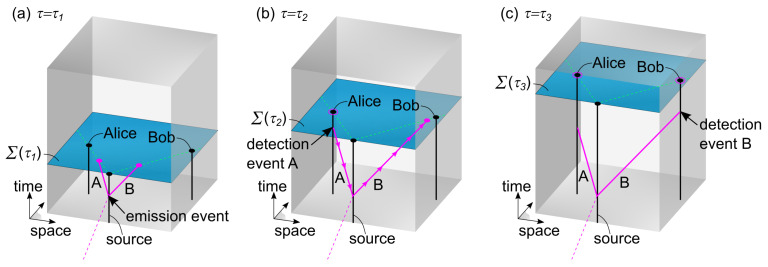
Illustration of an EPR experiment in the 4+1 formalism. In (**a**), at $\tau_{1}$, two entangled photon worldlines *A* and *B* are created in an emission event. In (**b**), at $\tau_{2}$, Alice has performed a measurement on photon *A*. This measurement produces a deterministic influence, which propagates along the photon worldlines as a function of τ, from Alice to Bob, as indicated by arrows. This influence affects both the past region of spacetime and spatially distant regions in the present. In (**c**), at $\tau_{3}$, Bob performs a measurement on photon *B*. In this way, the outcome of Bob’s measurement depends on the outcome of Alice’s measurement, even though his measurement occurs in a spatially distant region.

Firstly, the basic principle of the proposed EPR model in the 4+1 framework is illustrated in Figure 2 at three values of the evolution parameter τ. In essence, two entangled photons described using worldlines *A* and *B* are produced in an emission event and are traveling towards the measurement stations of Alice and Bob (see Figure 2a). When Alice performs a measurement on photon *A*, depending on the chosen setting for her PBS, the photon either passes or is deflected. As a consequence of Alice’s measurement, an influence travels backwards in the time dimension along worldline *A*, to the emission event, and then along worldline *B* as a function of τ (indicated by arrows in Figure 2b). In this way, influences can travel along worldlines to past regions of spacetime and to spatially distant regions in the present. Therefore, when Bob performs his measurement on photon *B*, his outcome will depend on Alice’s outcome (see Figure 2c).

**Figure 3:**
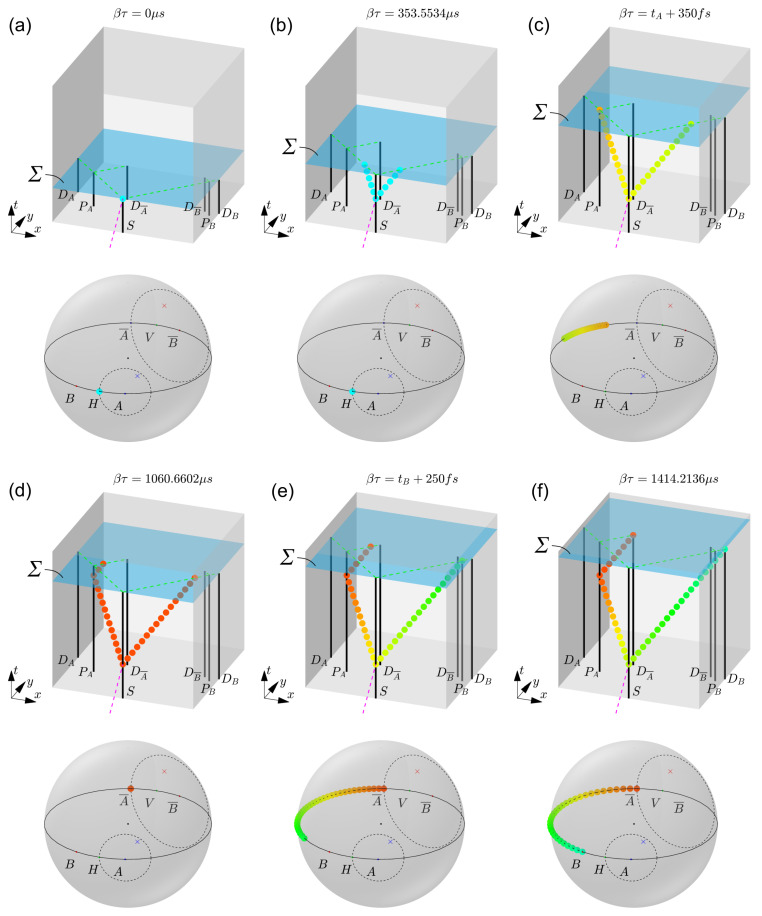
Simulation result of the EPR model in the 4+1 formalism, for the case of an initial state ${|H\rangle }_{A}{|H\rangle }_{B}$ of the entangled photons *A* and *B*. At six increasing values of τ (**a**–**f**), the spacetime configuration of worldlines is shown (top) and the polarization states along the photon worldlines are indicated on the Poincaré sphere (bottom). In (**a**,**b**), entangled photons *A* and *B* are created and travel, respectively, towards Alice and Bob. In (**c**), photon *A* interacts with PBS $P_{A}$, inducing a change in the polarization state across both entangled worldlines. Since hidden variable ${\mathrm{HV}}_{A}$ (blue cross) is located inside the circle around *A* on the Poincaré sphere, the polarization state of photon *A* is attracted to the state $\bar{A}$. In (**d**), both photons have collapsed to the state $\bar{A}$, and photon *A* is deflected towards detector $D_{\bar{A}}$. In (**e**), photon *B* interacts with PBS $P_{B}$. Since hidden variable ${\mathrm{HV}}_{B}$ (red cross) is inside the circle around $\bar{B}$, photon *B* is attracted to the state *B* on the Poincaré sphere. In (**f**), the final configuration is shown when photons *A* and *B* are detected, respectively, by $D_{\bar{A}}$ and $D_{B}$, leading to an outcome $\left(\bar{A},B\right)$. Similar results can be obtained for the initial state ${|V\rangle }_{A}{|V\rangle }_{B}$, for different settings of the polarizing beamsplitters, or for different choices of the hidden variables.

Next, a more detailed EPR model in the 4+1 formalism is developed, and simulation results are shown in Figure 3. As explained in Section 2.1, a preferred frame is considered in which at each value of τ the hypersurface Σ(τ) is characterized by a constant time $t_{e}=\beta \tau$, with positive scalar β. When assigning a unit $s^{*}$ to τ, this means that β has the unit of $s/s^{*}$. For simplicity, we choose $\beta =1s/s^{*}$. In this setting, each spacetime associated with a value of τ may equally be identified by the time $t_{e}=\beta \tau$ expressed in seconds. The polarizing beamsplitters $P_{A}$ and $P_{B}$ are positioned, respectively, at distances $d_{P_{A}}$ and $d_{P_{B}}$ from the source *S*. There are four detectors in total, $D_{A}$ and $D_{\bar{A}}$ for Alice and $D_{B}$ and $D_{\bar{B}}$ for Bob. Their positions are chosen such that the total path length $d_{tot}$ from source to each detector is the same: $d_{SA}=d_{S\bar{A}}=d_{SB}=d_{S\bar{B}}\equiv d_{tot}$. In Figure 3, $d_{P_{A}}$=275.77 km, $d_{P_{B}}$=381.84 km, and $d_{tot}$= 424.26 km, such that the time of flight for both photons is 1.414 ms. For six increasing values of τ, the worldline configuration in spacetime is depicted (top) and the polarization state along the worldlines is represented on the Poincaré sphere (bottom).

We further restrict the analysis to the case in which two entangled photons are created corresponding to the state ${|H\rangle }_{A}{|H\rangle }_{B}$ at βτ=0 μs (see Figure 3a). On the Poincaré sphere, both photons then correspond to the state *H*. The other case of an initial state ${|V\rangle }_{A}{|V\rangle }_{B}$ can be treated in an analogous way. Two local hidden variables, ${\mathrm{HV}}_{A}$ and ${\mathrm{HV}}_{B}$, which are defined as random points with a uniform distribution on the Poincaré sphere (respectively, the blue and red crosses in Figure 3), are invoked to determine the outcome of each experiment.

At βτ=353.6 μs, the two entangled photons can be seen traveling under a 90° spatial angle towards Alice and Bob (see Figure 3b). For each event on the photon worldlines *A* and *B* characterized by the affine parameter λ, a polarization state P(λ) is defined by a point on the Poincaré sphere. For simplicity, the affine parameter λ is chosen to be the 4D Cartesian distance $\sqrt{dx^{2}+dy^{2}+dz^{2}+{\left(cdt\right)}^{2}}$ in the preferred frame along the photon worldline, starting at the interaction event near Alice and ending at the end event near Bob. The following evolution law is proposed for the polarization state:

(3) $$\frac{dP(\lambda )}{d\tau }=K\frac{d^{2}}{d\lambda^{2}}P\left(\lambda \right)$$

where *K* is a constant determining the strength of the fundamentally local interaction. Here, a value $K=5\times 10^{10}$$s^{*-1}$ is chosen. To visualize the polarization state in Figure 3, at certain discrete events, circles are shown with hues corresponding to the azimuth angle, following the HSL color system.

At $\beta \tau \equiv t_{A}=919.24$ μs, the first photon reaches the PBS $P_{A}$ of Alice. The interaction between photon *A* and PBS $P_{A}$ imposes a condition on the polarization state at this interaction event (replacing Equation (3) at this event), governed via:

(4) $$\frac{dP(\lambda )}{d\tau }=-K^{*}\mathrm{sgn}\left[\left({\mathrm{HV}}_{A}-P\left(\lambda \right)\right)\cdot P_{A}\right]\left(P\left(\lambda \right)\times (P_{A}\times P\left(\lambda \right))\right)$$

where $K^{*}=10^{10}$$s^{*-1}$ is chosen. Equation (4) ensures that the polarization state at this event is attracted towards either state $P_{A}$ or state $P_{\bar{A}}$ on the Poincaré sphere, with the physical consequence that the photon is, respectively, passing through or is deflected by the PBS. The outcome is decided by the factor $-\mathrm{sgn}\left[\left({\mathrm{HV}}_{A}-P\left(\lambda \right)\right)\cdot P_{A}\right]$ in Equation (4). If ${\mathrm{HV}}_{A}$ is situated outside of a circle centered around $P_{A}$ passing through the state P(λ), then this factor is +1 and the outcome of the interaction is *A*. In the opposite case that ${\mathrm{HV}}_{A}$ lies inside of this circle, the factor is −1 and the outcome is $\bar{A}$. In Figure 3c, the latter case is illustrated at an intermediate value $\beta \tau =t_{A}+35$ fs during the transition from state *H* to state $\bar{A}$. Notice how the rest of the worldline gradually adjusts to this boundary condition as a consequence of Equation (3).

At about $\beta \tau =t_{A}+100$ fs, the complete worldline has aligned with the state $\bar{A}$, so we can say that both photons *A* and *B* have collapsed to the outcome of Alice. As τ increases further, photon *A* is deflected by PBS $P_{A}$ towards detector $D_{\bar{A}}$, while photon *B* continues on its path towards PBS $P_{B}$. This situation around βτ=106 μs is illustrated in Figure 3d.

At $\beta \tau \equiv t_{B}=1272.80$ μs, photon *B* arrives at PBS $P_{B}$ and the following evolution law is imposed at this interaction event:

(5) $$\frac{dP(\lambda )}{d\tau }=-K^{*}\mathrm{sgn}\left[\left({\mathrm{HV}}_{B}-P\left(\lambda \right)\right)\cdot P_{B}\right]\left(P\left(\lambda \right)\times (P_{B}\times P\left(\lambda \right))\right)$$

Since in this example the hidden variable ${\mathrm{HV}}_{B}$ is located inside the circle centered around $P_{\bar{B}}$ passing through $P_{\bar{A}}$, the state of photon *B* at the interaction event with PBS $P_{B}$ is forced towards $P_{B}$. In Figure 3e, the situation is shown at $\beta \tau =t_{B}+25$ fs, halfway through the transition of photon *B* towards state $P_{B}$. This means that photon *B* passes straight through PBS $P_{B}$ towards detector $D_{B}$.

Finally, Figure 3f shows the situation when photon *A* arrives at detector $D_{\bar{A}}$ and photon *B* at detector $D_{B}$, corresponding to an outcome $\left(\bar{A},B\right)$. A similar reasoning can be made for all other combinations of the initial state, polarizer settings, hidden variables, and different spatial geometries.

It can be readily verified that this scheme results in the desired quantum correlations given in Equation (2). If the initial state of photon *A* is ${|H\rangle }_{A}$ (in 50% of the cases), the probability for an outcome *A* corresponds to the chance of finding the uniformly distributed hidden variable ${\mathrm{HV}}_{A}$ outside of a circle on the Poincaré sphere with cone angle $2\theta_{A}$ around state *A*. This chance can be calculated as $\int_{2\theta_{A}}^{\pi }\int_{0}^{2\pi }\sin(\theta )d\theta d\phi /4\pi =\cos^{2}\left(\theta_{A}\right)$. Similarly, the chance for outcome $\bar{A}$ is $\sin^{2}\left(\theta_{A}\right)$. If the initial state of photon *A* is ${|V\rangle }_{A}$ (in the other 50% of the cases), then the chances of finding *A* or $\bar{A}$ are, respectively, $\sin^{2}\left(\theta_{A}\right)$ and $\cos^{2}\left(\theta_{A}\right)$. Together, this leads, as expected, to a 50% chance for *A* and 50% chance for $\bar{A}$. Right after Alice’s measurement, photon *B* collapses to the same state as photon *A* (which can have outcome *A* or $\bar{A}$). Assuming outcome $\bar{A}$ for Alice, the probability for outcome *B* for Bob (i.e., passing through the PBS) corresponds to the chance of finding the uniformly distributed hidden variable ${\mathrm{HV}}_{B}$ outside of a circle with opening angle $2\theta_{\bar{A}}-2\theta_{B}=\left(2\theta_{A}+\pi \right)-2\theta_{B}$ around state *B*. Or similarly, this corresponds to the chance of finding ${\mathrm{HV}}_{B}$ inside of the circle with cone angle $2\theta_{A}-2\theta_{B}$ around the state $\bar{B}$. This chance can be calculated as $\int_{0}^{2\theta_{A}-2\theta_{B}}\int_{0}^{2\pi }\sin(\theta )d\theta d\phi /4\pi =\sin^{2}\left(\theta_{A}-\theta_{B}\right)$. The overall probability of finding outcome $\left(\bar{A},B\right)$ must take into account the 50% chance of getting state $\bar{A}$ to begin with, resulting in $P\left(\bar{A},B\right)=\sin^{2}\left(\theta_{A}-\theta_{B}\right)/2$, in agreement with the quantum prediction of Equation (2). For all other combinations, the resulting correlations also agree with Equation (2).

In the simulation of Figure 3, the duration of the collapse of the polarization state of photon *B* in the preferred frame is about 100 fs. This is ten orders of magnitude faster than the time (1.6 ms) needed for light to travel between PBS $P_{A}$ and PBS $P_{B}$ and much faster than the lower limit of around 10,000*c* established using EPR tests [40]. If needed, *K* and $K^{*}$ can be made arbitrarily large to achieve a quasi-instantaneous collapse.

Not only does this EPR model reproduce the desired quantum correlations; it also highlights some important features of the 4+1 formalism. Firstly, it is a fundamentally realist model, since it is based on really existing worldlines in a τ-dynamic spacetime. Secondly, it is fundamentally deterministic, since all interactions and dynamics are governed with differential equations orchestrated using the evolution parameter τ. And thirdly, the model is fundamentally local since each polarization state only depends on neighboring polarization states or on local interactions with a PBS. So, even though EPR correlations appear nonlocal by an observer interpreting the outcomes in ordinary spacetime, these correlations originate from fundamentally local processes at a deeper level of reality in the 4+1 formalism.

### 3.2. Quantum Measurement and the Arrival Time Problem in the 4+1 Formalism

To illustrate problems with quantum measurement, the arrival time, and momentum conservation in standard quantum theory, we consider the isotropic emission of a particle from a point source and its detection with two opposing hemispherical screens with different radii. For simplicity, the particle is emitted in a short pulse, resulting in a propagating wave function in the form of a thin spherically symmetrical shell. According to quantum theory, in good approximation, there is a 50% chance of detecting the particle on the closest detector D1 and 50% chance on the farthest detector D2. When the wave function pulse passes detector D1 at time $t_{1}$, a partial collapse occurs in which it is decided if the particle is detected or not. In the case of detection, all momentum is transferred to detector D1, while an empty wave function travels further with no particular effect on detector D2. In the opposite case, there is no effect on detector D1, but then with 100% certainty, the particle will be measured by detector D2 at time $t_{2}$.

This experiment highlights a number of problems of standard quantum theory. Firstly, the measurement of the particle by detector D1 (with 50% chance of detection) at $t_{1}$ seems to have instantaneous consequences for the remaining, spatially separated part of the wave function, which is seemingly in conflict with relativity. This argument was first given by Einstein in his thought experiments called Einstein’s Boxes [41]. It led Einstein to conclude that it may be useful to consider actual particle positions: “It seems to me that this difficulty cannot be overcome unless the description of the process in terms of the Schrödinger wave is supplemented by some detailed specification of the localization of the particle during its propagation” [28]. Moreover, the collapse of the wave function at $t_{1}$ is troubled by the violation of momentum conservation, as was already argued by Einstein in 1905: “In accordance with the assumption to be considered here, the energy of a light ray spreading out from a point source is not continuously distributed over an increasing space but consists of a finite number of energy quanta which are localized at points in space, which move without dividing, and which can only be produced and absorbed as whole units” [42]. Hence, when assuming here that the isotropic wave function is associated with an isotropic momentum distribution, this leads to a conflict with conservation of momentum in the process of collapse when a measurement reveals the full particle momentum at detector D1 (or at D2). Bohmian mechanics has attempted to solve this problem by adding a hidden variable in the form of a particle position [43]. In the context of the present isotropic wave function, such a strategy indeed works, but, as will be discussed in Section 4, in other situations Bohmian mechanics does not offer a satisfying explanation for momentum conservation in free space [44].

Secondly, by choosing the two detectors at different distances from the source, additional problems with time are highlighted. The so-called arrival time problem is a fundamental problem in quantum mechanics of defining the time that a particle is detected at a known position [45,46,47]. And there are also other conceptual issues related to partial collapse at $t_{1}$ and the resulting empty or full wave functions [44,48]. For example, the fact that the wave function can interact with detector D1 without setting it off, or that the continuing wave function then always leads to detection at D2, is hard to grasp in standard quantum mechanics. Again, this becomes much easier when assuming that there is a hidden property pointing to a specific particle location.

**Figure 4:**
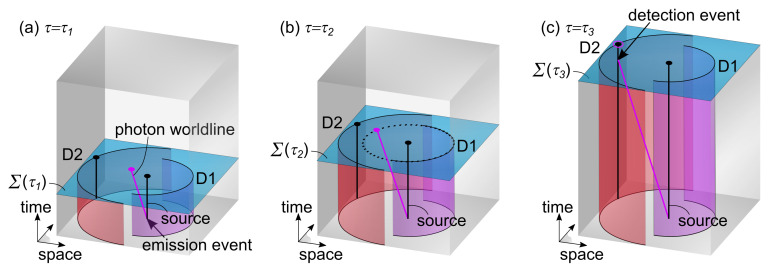
Illustration of a point source with two detectors in the 4+1 formalism (only a 2D spatial slice is shown). In (**a**), at $\tau =\tau_{1}$, a particle worldline carrying all momentum is created in an emission event, moving in a random spatial direction. In (**b**), at $\tau =\tau_{2}$, measurement by detector D1 only happens upon direct interaction between the particle worldline and the detector. Here, the particle worldline passes by without being detected. In (**c**), at $\tau =\tau_{3}$, the particle worldline interacts with a particular detector worldline, setting off detector D2.

Within the 4+1 formalism, we can propose a simple model for the same experiment (see Figure 4). Here, it is assumed that a single worldline carrying all the momentum of the particle is emitted in a random direction (see Figure 4a). This worldline keeps its initial spatial orientation but simply grows as Σ(τ) shifts in the positive time dimension. Considering the simplicity of this model, no further evolution laws are needed. At $\tau_{2}$, it becomes very clear that a measurement via detector D1 only occurs if the worldline interacts directly with this detector (see Figure 4b). If not, the worldline continues until it unavoidably interacts with detector D2 (see Figure 4c). As a result, this simple model reproduces the expectation from quantum mechanics. In addition, it highlights the advantage of using worldlines in the 4+1 formalism for avoiding problems with quantum measurement. Namely, by relying on a single worldline that determines the outcome of the experiment, there is no measurement, collapse, or arrival time problem. And by insisting that this worldline is a geodesic between key interaction events, there is no conflict with conservation of momentum.

### 3.3. Interference, Wave–Particle Duality, and Superposition in the 4+1 Formalism

The quantum superposition of a particle is yet another key feature of quantum mechanics, with relevance to planned tests of quantum gravity in the laboratory. Here, a standard interferometry setup (Mach–Zehnder-type) with 50/50 beamsplitters is considered in which a photon is brought in a spatial superposition. We begin by repeating the standard quantum treatment of such interferometer. At the input of the interferometer, we consider the state $|\Psi \rangle$ representing the polarization state of the incident photon. The two beamsplitters BS1 and BS2 are represented in the Jones matrix formalism by [49]:

(6) $$BS1=BS2=\frac{1}{\sqrt{2}}\left[\begin{array}{cc} -1 & 1\\ 1 & 1 \end{array}\right]$$

Hence, the quantum state after BS1 is given by:

(7) $$|\Psi_{1}\rangle =\left(-\frac{1}{\sqrt{2}}|A\rangle +\frac{1}{\sqrt{2}}|B\rangle \right)|\Psi \rangle$$

where $|A\rangle$ represents the spatial state of branch A after reflection under a 90° spatial angle, $|B\rangle$ represents branch B after transmission, and $|\Psi \rangle$ still captures the polarization state. Furthermore, in branch A, an excess path length can be introduced, which adds a phase ϵ to this path. As a result, the state becomes:

(8) $$|\Psi_{2}\rangle =\left(-\frac{1}{\sqrt{2}}e^{i\epsilon }|A\rangle +\frac{1}{\sqrt{2}}|B\rangle \right)|\Psi \rangle$$

From Equation (8), we find, as expected, that the probability of measuring the photon in branch A or B (if a detector was placed there) is given by:

(9) $$\begin{array}{c} Q_{A}=\frac{1}{2}\\ Q_{B}=\frac{1}{2} \end{array}$$

After BS2, the state can be rewritten in terms of the output ports $|C\rangle$ and $|D\rangle$:

(10) $$|\Psi_{3}\rangle =\left(-\frac{1}{2}e^{i\epsilon }|\Psi \rangle +\frac{1}{2}|\Psi \rangle \right)|C\rangle +\left(-\frac{1}{2}e^{i\epsilon }|\Psi \rangle -\frac{1}{2}|\Psi \rangle \right)|D\rangle$$

Detectors D1 and D2 only detect the component of the total state, respectively, in branches *C* and *D*. The quantum states arriving at these detectors are then given by:

(11) $$\begin{array}{c} |\Psi_{C}\rangle =-\frac{1}{2}e^{i\epsilon }|\Psi \rangle +\frac{1}{2}|\Psi \rangle \\ |\Psi_{D}\rangle =-\frac{1}{2}e^{i\epsilon }|\Psi \rangle -\frac{1}{2}|\Psi \rangle \end{array}$$

Finally, this leads to the following expectation values at the detectors:

(12) $$\begin{array}{c} Q_{C}=\frac{1}{2}-\frac{1}{2}\cos\left(\epsilon \right)\\ Q_{D}=\frac{1}{2}+\frac{1}{2}\cos\left(\epsilon \right) \end{array}$$

Two remarks can be made about this standard quantum result that are relevant for further analysis. Firstly, the probability of measuring a photon at one of the detectors (D1 or D2) depends on both branches of the interferometer. This follows from the presence of ϵ in Equation (12). And it reveals an interference effect or wave-like behavior. Yet, each individual photon is only detected by one of both detectors, demonstrating particle-like behavior. This constitutes the well-known wave–particle duality. Secondly, quantum theory does not specify along which branch a particle actually travels but assumes instead that the particle ends up in a superposition of going either way. At present, it is not understood what the gravitational effect is of a particle in such a spatial superposition, since this requires a theory of quantum gravity. For example, it is not known from any experiment if the gravitational field also ends up in a superposition (if such a thing is possible) or if it behaves in some kind of classical way.

**Figure 5:**
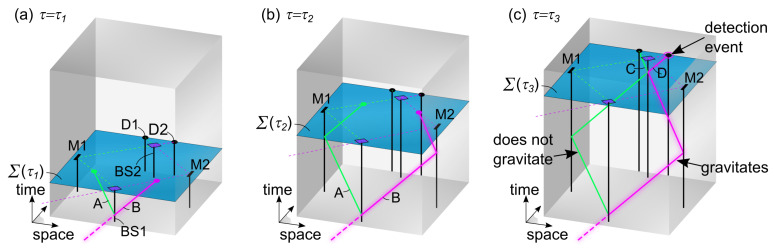
Illustration of a Mach–Zehnder interferometer in the 4+1 formalism. In (**a**), all beamsplitters, mirrors, and detectors are shown (only the phase delay element is not drawn). Each incident photon consists of a bundle of *N* particle worldlines. After passing beamsplitter BS1, these worldlines split up into branches A and B. Only one branch contains the momentum-carrying worldline (magenta); the other branch does not carry momentum (green). In (**b**), the particle worldlines reflect at mirrors M1 and M2, but only the momentum worldline in the magenta channel transfers recoil momentum to M2. In (**c**), the particle worldlines interact with beamsplitter BS2 and split up into channels C and D. The momentum worldline determines which detector clicks. Spatial reorganization of worldlines is determined via evolution laws in a fundamentally local and deterministic way as a function of τ. Only one branch of the interferometer actually gravitates, so there is no superposition of gravitating configurations.

Next, a model for the Mach–Zehnder interferometer is developed within the 4+1 formalism (see Figure 5). The interferometer setup is, for simplicity, chosen stationary in the preferred frame. Similar as for the EPR experiment, Σ(τ) identifies a single time coordinate $t_{e}=\beta \tau$ for each value of τ.

Before entering the interferometer, each incident photon is modeled as a bundle of *N* (a large number) worldlines (null geodesics) called particle worldlines, labeled with index i=1:N. Only one worldline (for which i=p), referred to as the momentum worldline, carries the energy and momentum of the photon. Each worldline of this incident bundle is further characterized by the same polarization and phase. As the evolution parameter τ increases, the hypersurface Σ(τ) shifts forward in the time dimension. Consequently, worldlines belonging to all optical components of the interferometer and particle worldlines of the photon, which have their endpoints on the hypersurface Σ(τ), are growing.

Once the particle worldlines enter the interferometer, there are a number of optical elements with which these worldlines can interact, identifying key interaction events of the form (x,y,z,t). For example, $e_{1}$ represents the interaction event with BS1, $e_{2}$ and $e_{3}$ are interaction events with the mirrors, respectively, in branches *A* and *B*, and $e_{4}$ is the interaction event with BS2. Between key interaction events, worldlines are assumed to be straight (null geodesics). Each worldline can follow either branch *A* or *B* between BS1 and BS2 and either branch *C* or *D* after BS2. Next, we elaborate how the *N* worldlines are split up along these possible paths.

Let us first analyze the case just after interaction with beamsplitter BS1, when the endpoints of the photon worldlines are situated just after BS1 (see Figure 5a). At each value of τ, quantum mechanics provides two quantum measures $M_{A}$ and $M_{B}$, respectively, at the endpoints of paths A and B on the hypersurface Σ(τ), which are proportional to the probabilities $Q_{A}$ and $Q_{B}$ of finding the particle, respectively, along paths A or B (see Equation (9)). In this simple geometry, these quantum measures can be established in a deterministic and local way along null geodesics following the path integral formalism. In the 4+1 framework, we are allowed to propagate this information backwards in time, respectively, along paths A and B in a fundamentally local way as a function of τ. We further assume that this information is transferred much faster as a function of τ compared to other relevant τ-dynamics. As a result, $M_{A}$ and $M_{B}$ become available information at the past interaction event $e_{1}$ almost immediately after the interaction with BS1 as a function of τ. Hence, at the interaction event $e_{1}$, there is enough information to spatially separate the *N* worldlines into two groups (along path A or path B) in a fundamentally local and deterministic way according to the following evolution law:

(13) $$\frac{dN_{A}}{d\tau }=-\frac{dN_{B}}{d\tau }=\xi \left(\frac{M_{A}}{N_{A}}-\frac{M_{B}}{N_{B}}\right)$$

Here, for simplicity, $N_{A}$ and $N_{B}$ are modeled as continuous variables representing approximately the discrete numbers of worldlines aligned, respectively, along the paths A and B. The initial numbers of worldlines just after the interaction event are assumed to correspond to the numbers of incident worldlines traveling in the same direction before the interaction, i.e., $N_{A}\left(\tau_{e_{1}}\right)=0$ and $N_{B}\left(\tau_{e_{1}}\right)=N$. Equation (13) implies that worldlines gradually shift between channels A and B as a function of τ, until equilibrium is reached. The τ-interval in which this reorganization occurs is set by the constant ξ, and this parameter is chosen sufficiently large that the reorganization occurs before any significant shift of Σ(τ) has occurred. Equation (13) leads to an equilibrium governed by:

(14) $$\frac{M_{A}}{N_{A}}=\frac{M_{B}}{N_{B}}$$

or equivalently, using the proportionality between $M_{A}$ and $Q_{A}$ and between $M_{B}$ and $Q_{B}$ (with same proportionality constant):

(15) $$\frac{N_{A}}{N_{B}}=\frac{Q_{A}}{Q_{B}}$$

Therefore, the fractions of worldlines taking paths A or B become equal to the fractions of the corresponding quantum expectation values from Equation (7).

If we assume that particle worldlines are randomly assigned to the two groups along branches A and B, then the probability that the momentum worldline labeled with index i=p travels along branch A or B is also proportional to the number of worldlines in these respective channels and thus also proportional to the quantum expectation value of each channel. Since only the momentum worldline can interact with a detector and set it off, this means that the chance of detecting the photon in channel A or B agrees with the quantum prediction. An important difference with the standard quantum description is that here a clear decision is made along which branch of the interferometer the particle momentum travels. If the momentum worldline follows branch *A*, a reflection occurs and the associated recoil momentum is transferred to the beamsplitter BS1. In the other case that the momentum worldline follows branch *B* (transmission), no momentum is transferred to BS1.

At larger values of τ but still before interacting with BS2, the two bundles along paths A and B interact with optical systems (mirrors and an optical element that introduces a phase delay) (see Figure 5b). This produces new interaction events without affecting the number of particle worldlines in branches A and B. Only the mirror that interacts with the momentum worldline actually receives a momentum recoil.

Next, we analyze what happens after interaction with beamsplitter BS2 (see Figure 5c). Similar reasoning as was made for branches A and B is followed here for branches C and D, leading to the following evolution law:

(16) $$\frac{dN_{C}}{d\tau }=-\frac{dN_{D}}{d\tau }=\xi \left(\frac{M_{C}}{N_{C}}-\frac{M_{D}}{N_{D}}\right)$$

with quantum measures $M_{C}$ and $M_{D}$ being proportional, respectively, to $Q_{C}$ and $Q_{D}$ from Equation (12) (with same proportionality constant). Hence, Equation (16) leads to the following equilibrium:

(17) $$\frac{N_{C}}{N_{D}}=\frac{Q_{C}}{Q_{D}}$$

Similar as explained above, this leads to an outcome of the Mach–Zehnder experiment in agreement with quantum mechanics. The momentum recoil with BS2 at the interaction event $e_{4}$ is determined by the chosen path of the momentum worldline. In the case that a reflection of the momentum worldline occurs, momentum is transferred to BS2. Otherwise, no momentum is transferred.

The above model for the Mach–Zehnder interferometer highlights some interesting features of the 4+1 formalism. Firstly, it shows how worldlines can be spatially arranged as a function of τ in a fundamentally local and deterministic way in order to reproduce quantum interference. Secondly, it shows how momentum transfer can be concentrated at local interaction events between the momentum worldline of the photon and worldlines of the interferometer, while in between interaction events the momentum worldline is a null geodesic that preserves momentum. Thirdly, it sheds light on wave–particle duality and superposition. Even though the model relies on many particle worldlines that pass along both branches A and B and that codetermine the output of the interferometer, only one of these worldlines actually carries the energy and momentum of the photon and can set off a detector. Therefore, here, a photon is envisioned as an ensemble of many worldlines that enable its wave-like features, but with only a single momentum-carrying worldline that enables its particle-like features. Whereas the standard concept of quantum superposition states that the photon ends up in a superposition of going either via branch A or via branch B, in the 4+1 formalism, one can say that some constituent worldlines of the photon do travel both ways but that the momentum-carrying worldline only takes a single path through the interferometer. In Section 4, further consequences for laboratory tests of quantum gravity are discussed.

## 4. Discussion

The theoretical models in Section 3 illustrate how EPR nonlocality, quantum measurement and the arrival time problem, and superposition can be understood in the 4+1 formalism. Furthermore, these models highlight several key features of the 4+1 formalism.

Firstly, the fact that the 4+1 formalism relies on worldlines rather than on wave functions or fields offers a number of advantages. By allowing only worldlines to set off detectors by means of local interactions, there is no measurement problem or arrival time problem. This has been emphasized in the model in Section 3.2. Local conservation of momentum is guaranteed by restricting momentum transfer to local interaction events between worldlines and by insisting on straight worldlines between interaction events, as is stressed in the interferometer model in Section 3.3. It is interesting to compare this with Bohmian mechanics [43]. Since Bohmian mechanics relies on particle trajectories in standard spacetime, it can also explain determinate outcomes. However, Bohmian mechanics is explicitly nonlocal, and its particle trajectories seem to violate local momentum conservation, for example, in the case of wiggling trajectories in a double-slit experiment [50] or in an interferometer with a removed second beamsplitter [51]. Another advantage of the chosen worldline-based approach is that it provides a straightforward explanation of the quantum-to-classical transition—the problem of where the boundary lies between the microscopic quantum world and the macroscopic classical world [4]. This is because the 4+1 framework is essentially a classical construction without quantum superpositions or Schrödinger cats at a fundamental level. Here, observations are the result of interactions between classical worldlines. There may be dynamics in the configuration of worldlines as a function of τ, but per definition our observations only capture the crystallized, classical configuration of worldlines that has reached an equilibrium. This explains our observation of a classical world. Hence, it is only the correlations between classical observations that may reveal a typical quantum character when analyzed from within ordinary spacetime. Note that the classical nature of the 4+1 formalism does not necessarily imply that reality must be classical altogether. Ultimately, more fundamental, discrete building blocks of τ-dynamic worldlines at the Planck scale may be required.

Secondly, the 4+1 formalism relies on two forms of time (a time dimension *t* and an external evolution parameter τ) and on a shifting hypersurface Σ(τ). As explained in Section 2.2 and Section 2.3, this construction allows observers to map out measured events in a standard 4D spacetime, as is desired from relativity. But it also explains our observation of the present and of the flow of time. This follows directly from the assumption that our observation of the present is physically linked to the hypersurface Σ(τ) at the interface between a disordered future region and a crystallizing past region. Notice that this may point to a form of criticality, namely that some of the most interesting phenomena happen at a boundary between chaos (the future region of spacetime) and order (the past, crystallized region of spacetime). Many theories have been developed that add spatial and/or time dimensions to 4D spacetime, such as string theory, brane worlds, and other multidimensional theories [52,53,54,55]. Some of these theories aim to solve the hierarchy problem, to merge quantum theory with gravity, to find a theory of everything, or to explain dark matter. However, a general problem with these theories is the lack of direct experimental evidence for extra dimensions [56,57]. As a result, much effort must be made to make these dimensions invisible for our observations. For example, the extra dimensions must be rolled up tightly or compactified, or particles must be confined to four-dimensional hypersurfaces within a larger-dimensional space. However, the 4+1 formalism does not suffer from this problem. In fact, it is argued that the extra form of time is necessary for explaining our observation of the flow of time. Also, many retrocausal theories (or future-input-dependent theories) within four-dimensional spacetime have been developed [29,38,39,58,59]. For example, it is known that relaxing measurement independence (and thus allowing future-input-dependence) is a powerful means of explaining EPR correlations [37,38,39]. However, a major problem with such theories is that retrocausality (or future-input-dependence) is incompatible with the causal structure of standard spacetime and leads to paradoxes [60]. To make such theories viable, one must explain how retrocausality really works. For example, to avoid paradoxes when considering the concept of influences traveling backwards in time, it seems that a second form of time is needed to distinguish different phenomena occurring at the same spacetime coordinates. The 4+1 formalism precisely does this: it explains in a fundamentally deterministic way how retrocausal phenomena may occur.

And thirdly, an important message of this work is that in the 4+1 formalism, the concepts of realism, locality, and determinism can be rescued at a more fundamental level of reality, whereas this is certainly hopeless within ordinary spacetime. This is relevant when valuing an intuitive, single-world view of reality. All models in Section 3 rely on fundamentally realist and fundamentally local interactions between or along worldlines occurring in a deterministic way as a function of τ. The most striking example of this is the EPR model in Section 3.1. This model shows how EPR nonlocality can be understood in a fundamentally realist, local, and deterministic way, in the spirit of Costa de Beauregard’s zigzag action along worldlines. And the interferometer model in Section 3.3 highlights how wave–particle duality and superposition can be understood by imagining each photon as a bundle of τ-dynamic worldlines of which only one triggers the detector. It is then only from the limited perspective of a standard observer, who interprets quantum results in standard four-dimensional spacetime, that the correlations between measured events require concepts like nonlocality, collapse, or superposition.

In future work, a more ambitious program for replacing the standard quantum formalism with a formalism of τ-dynamic worldlines can be undertaken. The three case studies in Section 3 only give a rough idea of what such a theory may look like. In this aspect, the present work shares the view of several physicists, like Einstein, de Broglie, Schrödinger, Penrose, ’t Hooft, Weinberg, Smolin, and Hossenfelder, that quantum theory may ultimately need to be replaced by a different theory. For example, ’t Hooft says: “What the Schrödinger equation is describing is not exactly what is happening; it merely describes the tip of a gigantic iceberg, in which most processes happen far beneath the waterline” [61]. Weinberg writes: “On the other hand, the problems of understanding measurement in the present form of quantum mechanics may be warning us that the theory needs modification” [62]. Smolin states: “Quantum weirdness isn’t real—We’ve just got space and time all wrong” and “To solve all these issues, we need to wipe the slate clean, go back to the first principles of quantum theory and general relativity, decide which are necessary and which are open to question, and see what new principles we might need” [63]. And lastly, Penrose points out: “My own view is that to understand quantum non-locality we shall require a radically new theory. This new theory will not just be a slight modification to quantum mechanics but something as different from standard quantum mechanics as General Relativity is different from Newtonian Gravity. It would have to be something which has a completely different conceptual framework. In this picture, quantum non-locality would be built into the theory” [64]. The theory presented in this work aims to achieve precisely what is suggested by many of these physicists: to physically explain what happens “below the surface” in a measurement by relaxing assumptions about spacetime and by replacing the quantum formalism.

The 4+1 formalism developed in this work is restricted to flat 4D spacetime. A logical further trajectory is to explore a similar 4+1 formalism based on curved 4D spacetimes that satisfy the Einstein field equations. A good way to summarize such an extended 4+1 concept is through the following modification of the Einstein field equations:

(18) $$G_{\mu \nu }\left(\tau \right)=\frac{8\pi G}{c^{4}}T_{\mu \nu }\left(\tau \right)$$

where *G* is the gravitational constant and *c* the speed of light and where the appearance of τ emphasizes that the energy–stress tensor $T_{\mu \nu }$ and the spacetime curvature expressed by $G_{\mu \nu }$ become τ-dependent objects. Establishing a connection between these curved spacetimes is much more challenging but may be realized, for example, with the procedures developed by Land [21,22,23]. Additionally, a more advanced evolution law for the hypersurface Σ(τ) in curved spacetime is needed, and worldlines must be geodesics between local interaction events in order to satisfy conservation of momentum. Furthermore, the above-mentioned worldline-based replacement for quantum theory should be extended towards curved spacetime. Together, such a generalized 4+1 formalism could enable a unified understanding of gravitational observations aligned with general relativity, quantum phenomena, and the passage of time, forming a compelling foundation for a theory of quantum gravity. Returning to the problem of time mentioned in the Introduction, it is not unexpected that a modification of the concept of time plays a pivotal role in achieving this unification.

There are also implications of the presented 4+1 formalism for laboratory tests of quantum gravity [65,66]. The interferometer model in Section 3.3 shows that there is only one classical, momentum-carrying worldline in each spacetime corresponding to a value of τ. This means that one may expect that the gravitational effect of the particle in the interferometer is also restricted to just one branch of the interferometer. And according to Equation (18), the gravitational effect of this branch is essentially classical. As a consequence, in low-energy, table-top tests of quantum gravity consisting, for example, of two gravitationally coupled interferometers, no gravitationally induced entanglement is expected. This is in contrast with the prevailing view that, if the considered particles end up in a superposition, the gravitational field must also end up in a superposition, resulting in gravitationally induced entanglement.

## 5. Conclusions

This work presents a 4+1 formalism aimed at addressing fundamental issues in quantum gravity. It incorporates a flat 4D spacetime evolving with τ, a hypersurface Σ(τ), and τ-dynamic worldlines. Within this framework, a model for an EPR experiment is developed elucidating quantum nonlocality in the 4+1 formalism. The quantum measurement and arrival time problems are analyzed by considering an isotropic source and hemispherical detectors. And a model for a Mach–Zehnder interferometer is developed to clarify particle–wave duality and superposition. These results showcase intriguing aspects of the 4+1 formalism. It can reproduce essential quantum phenomena without suffering from the measurement problem, satisfies local conservation of momentum, and offers an explanation for the quantum-to-classical transition. By relying on two forms of time and a shifting hypersurface, it enables us to simultaneously understand observations of a 4D spacetime and of the flow of time. And it upholds principles like locality, realism, and determinism at a fundamental level. With these properties, the 4+1 formalism produces an intuitive, single-world view of reality and forms an intriguing basis for a theory of quantum gravity.

## Data Availability

Not applicable.
